# miR-3187-3p enhances migration and invasion by targeting PER2 in head and neck squamous cell carcinomas

**DOI:** 10.7150/jca.58593

**Published:** 2021-06-26

**Authors:** Lei Xiao, Chao Liu, Shuiting Zhang, Yuanzheng Qiu, Donghai Huang, Diekuo Zhang, Huihong Chen, Hang Ling, Yong Liu, Xin Zhang

**Affiliations:** 1 Department of Otolaryngology Head and Neck Surgery, Xiangya Hospital, Central South University, 87 Xiangya Road, Changsha, Hunan 410008, China.; 2 Otolaryngology Major Disease Research Key Laboratory of Hunan Province, 87 Xiangya Road, Changsha, Hunan 410008, China.; 3 Clinical Research Center for Pharyngolaryngeal Diseases and Voice Disorders in Hunan Province, 87 Xiangya Road, Changsha, Hunan 410008, China.; 4 National Clinical Research Center for Geriatric Disorders (Xiangya Hospital), 87 Xiangya Road, Changsha, Hunan 410008, China.

**Keywords:** Head and neck squamous cell carcinoma, miR-3187-3p, invasion, metastasis

## Abstract

Invasion and metastasis are major contributors to treatment failure in patients with head and neck squamous cell carcinomas (HNSCCs) and microRNAs (miRNAs) are reported to play important roles in tumor progression. Our study therefore try to find the crucial miRNAs and reveal their molecular and functional mechanisms involved in migration and invasion of HNSCCs. Through The Cancer Genome Atlas (TCGA) data analysis, we screened out miR-3187-3p and its biological function and specific mechanism were further analyzed. The wound-healing and transwell invasion assay demonstrated that miR-3187-3p promoted the capacity of migration and invasion of HNSCCs *in vitro*. Luciferase reporter assays showed that PER2 was a direct target of miR-3187-3p, which could reverse the effect of miR-3187-3p in HNSCCs. Furthermore, we found that miR-3187-3p / PER2 axis activated the Wnt / β-catenin signaling pathway in HNSCCs. Altogether, our study indicated that miR-3187-3p enhanced migration and invasion by targeting PER2 in HNSCCs.

## Introduction

Head and neck squamous cell carcinomas (HNSCCs), derived from the epithelial cells of the upper aerodigestive tract (the oral cavity, the nasopharynx, the oropharynx, the hypopharynx and the larynx) 1, have approximately 800,000 new cases yearly with more than 50% 5-year mortality 2. Despite recent advances in treatment strategy, especially targeted therapy 3 and checkpoint therapy 4, the prognosis for patients with HNSCCs has remained uniformly poor, which is definitely due to invasion and metastasis of tumor for a large part 5. It is therefore important and necessary to reveal the molecular mechanisms underlying invasion and metastasis of HNSCCs, which can offer vital countermeasures and predicted biomarkers for the improvement of prognosis of patients with HNSCCs.

First discovered in 1993 6, microRNAs (miRNAs) are a family of ~22 nucleotide (nt) long small non-coding RNAs that function by silencing their target gene 7. It is clear now that miRNAs are involved in the regulation of most protein-coding transcripts activities, contributing to the normal cellular and physiological function of human beings 8. Meanwhile, the aberrant expression of miRNAs inevitably could regulate diverse malignant biological behavior, including proliferation, metastasis, radioresistance, immunosuppression and so on 9-14. A mass of researches have revealed that miRNAs dysregulation can contribute to tumor progression in numerous kinds of cancers, including HNSCCs 15, 16. Hence adjustment of specific dysregulation miRNAs represents a promising treatment avenue toward HNSCCs 8.

Previously, through analyzing The Cancer Genome Atlas (TCGA) database, we had screened a number of differentially expressed miRNAs associated with metastasis of HNSCCs, in which miR-3187-3p was included 16. miR-3187-3p was reported to exert tumor-promoting role in colorectal cancer and non-small cell lung cancer 17, 18. However, the exact role of miR-3187-3p in HNSCC remains to be elucidated. In this study, we demonstrated that miR-3187-3p was not only an independent prognostic factor for HNSCCs patients but also tightly associated with migration and invasion traits of HNSCCs by targeting PER2 and activating the Wnt / β-catenin signaling pathway. These findings provide valuable clues to understand the molecular mechanisms involving migration and invasion of HNSCCs.

## Materials and methods

### Cell culture

In the study, four HNSCCs cell lines JHU011, Tu686, Tca8113, Fadu and one precancerous lesions of the oral mucosa cell line DOK were used. JHU011 cell line were kindly gifted by Dr Joseph Califanio (University of California, San Diego, USA). Tu686 cell line was kindly offered by Dr Zhuo G. Chen (Emory University Winship Cancer Institute, USA) 16. Tca8113 and DOK cell lines were acquired from The Cell Bank of Type Culture Collection of Chinese Academy of Sciences. Fadu cell line was bought from ATCC. These cells were maintained in appropriate medium (DMEM/F12 medium, DMEM basic medium, or RPMI 1640 medium) supplemented with 10% fetal bovine serum (FBS, Biological Industries) in 5% CO_2_ at 37 °C.

### Patient samples

All HNSCCs tissue samples and patients' information were obtained in the same manner as our previous study 16, in which 83 cases of HNSCCs were included. The study was conducted in accordance with the Declaration of Helsinki and approved by the Research Ethics Committee of Xiangya hospital, Central South University, and written informed consents were obtained from all these patients.

### RNA isolation and gene expression analysis

Total RNA was extracted using Trizol reagent (Invitrogen). First strand cDNA was synthesized with the All-in-One^TM^ cDNA synthesis kit (GeneCopoeia). Quantitative real-time PCR (qRT-PCR) was conducted with QuantStudio 7 Flex Real-Time PCR System (ThermoFisher Scientific). Data derived from qRT-PCR were caculated using 2^-ΔΔCt^ method and normalized to U6 for miRNA or GAPDH for mRNA. The experiments were carried out for three times to get the final average result. The primers used are listed in Supplementary Table S1.

### Western blotting

Total protein was obtained using RIPA lysis buffer. Protein concentration was quantified by BCA assay. Then homogenates containing 20 µg protein in SDS-PAGE sample loading buffer were loaded on an 8-12% SDS-PAGE gels and protein were transferred to PVDF membranes after electrophoresis. Next, the membrane was blocked with NcmBlot blocking buffer (NCM Biotech, Suzhou, China) for 10min and subsequently incubated with relevant primary antibodies overnight at 4 °C, followed by incubation with corresponding secondary antibody for 50 min at room temperature. Finally, target protein was detected using ECL substrates.

### Transfection

Fadu and Tca8113 cell lines were transfected with miR-3187-3p mimics / negative control (NC) (Genepharma, Suzhou, China) following the manufacturer's protocol. JHU011 and Tu686 cell lines were transfected with miR-3187-3p inhibitors / NC (RiboBio, Guangzhou, China) according to the manufacturer's guidelines. PER2 overexpression plasmid transfection was performed using FuGENE HD Reagent (Promega).

### Wound-healing and transwell invasion assay

The wound was scratched using a 200 μL sterile micropipette tip when the HNSCCs cells reached near 100% confluence. The cells were continued to be cultured for 24 - 96 h without serum. The scratch wound was photographed under phase-contrast microscope, and the scratch area was assessed by ImageJ software.

As to transwell invasion assay, 150 μL cell suspension (1.5-2×10^4^) of serum-free medium were seeded in the top Transwell chamber, while 500 μL complete medium containing 10% FBS was added in the lower chamber. After 48 h incubation, non-invaded cells were erased and the invaded cells were fixed and stained, and the number of invaded cells were then counted in five randomly selected area under high magnification microscope (200 ×).

### Luciferase reporter assays

Luciferase assays were conducted 48 h after co-transfection with miR-3187-3p mimic or NC in combination with wild or mutated type PER2 plasmids (GeneCopoeia) in Fadu cells. Relative luciferase activity was accessed by normalizing the Firefly luciferase with the Renilla luciferase values using the Dual-Luciferase Reporter Assay System (Promega).

### Statistical analysis

Statistical analysis was performed with SPSS21.0. Descriptive statistics were presented as the means ± SD. Data were analyzed using appropriate statistical methods including independent t tests, Wilcoxon tests and ANOVA. Kaplan-Meier method was used to plot survival curves and compared by log-rank test. P < 0.05 was considered as statistically significant.

## Results

### Clinicopathologic features of miR-3187-3p in HNSCCs

**Figure 1:**
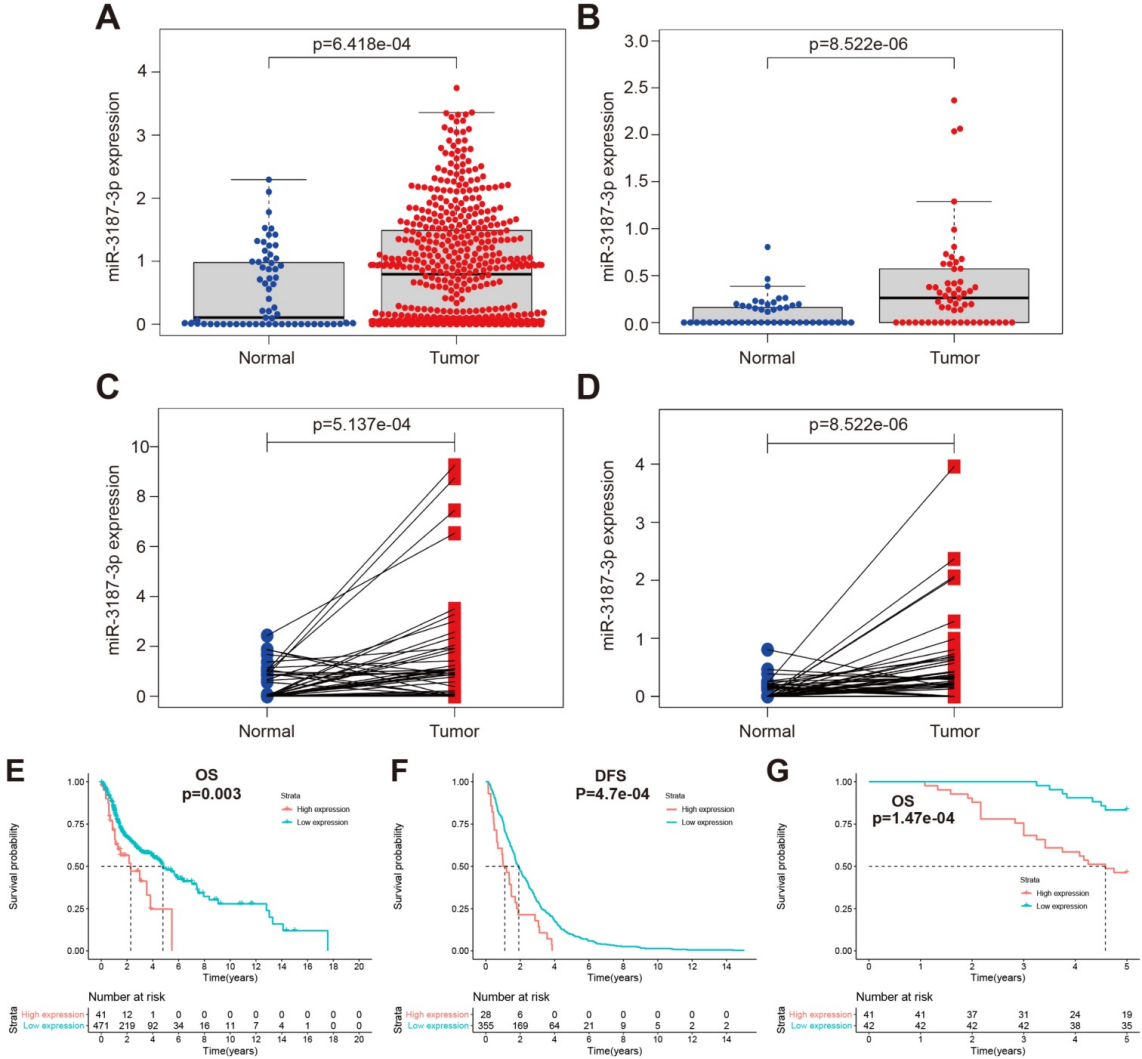
** Clinicopathologic features of miR-3187-3p in HNSCCs. A.** The expression of miR-3187-3p in 525 HNSCCs samples and 44 normal sample in TCGA database. **B.** The expression of miR-3187-3p in 57 laryngeal squamous cell carcinoma (LSCC) samples and 57 adjacent normal sample in GEO database (GSE133632). **C.** The expression of miR-3187-3p in 43 paired TCGA tumor and corresponding adjacent noncancerous tissues. **D.** The expression of miR-3187-3p in LSCC tissues (57 samples) and paired adjacent normal mucosa tissues in GEO database (GSE133632). **E, F.** Overall survival (OS) (E) and Disease-free survival (DFS) (F) analysis was implemented in patients with HNSCCs according to the expression level of miR-3187-3p using Kaplan-Meier survival curves in TCGA data (The best cutoff was obtained through Xtile to distinguish high and low miR-3187-3p levels; cutoff = 3.9).** G.** Kaplan‐Meier analysis in patients with HNSCC between the high expression group (n = 41) and low expression group (n = 42) of miR-3187-3p.

In our previous study, we had identified aberrant expression profile of miRNAs associated with HNSCCs metastasis, in which miR-3187-3p was included 16. To further investigate the clinical significance of miR-3187-3p in patients with HNSCCs, the relative expression of miR-3187-3p was assessed in TCGA and GEO database using R package limma. miR-3187-3p was highly expressed in 525 HNSCCs samples from TCGA and 57 HNSCCs samples form GEO (GSE133632) compared with the adjacent normal tissues (Fig. 1A, B; P < 0.001). Paired cancer and corresponding paracancerous tissue in both TCGA and GEO also showed a significantly higher expression of miR-3187-3p in tumor (Fig. 1C, D; P < 0.001). Furthermore, miR-3187-3p was strongly associated with both clinical and pathologic N Status (Table 1; P < 0.001) and survival analysis revealed that HNSCCs patients with higher miR-3187-3p expression displayed a poorer prognosis than those with lower expression of miR-3187-3p (Fig. 1E, F; P < 0.05).

Besides, we collected 83 tissue samples to further analyze the clinical significance of miR-3187-3p in HNSCCs and the relative expression of miR-3187-3p was quantified by qRT-PCR. As shown in Table 2, the high expression of miR-3187-3p was closely related to the higher T stage, advanced clinical stage and lymph node metastasis in HNSCCs patients (Table 2; P <0.05). Meanwhile, the prognosis of patients with high miR-3187-3p expression was significantly worse than those with low miR-3187-3p expression (Figure 1G, P < 0.001), which was consistent with the results of TCGA and GEO database analysis. Collectively, these data suggest that miR-3187-3p may be a valuable prognosis biomarker for patients with HNSCCs.

### Overexpression of miR-3187-3p promotes HNSCCs migration and invasion

**Figure 2:**
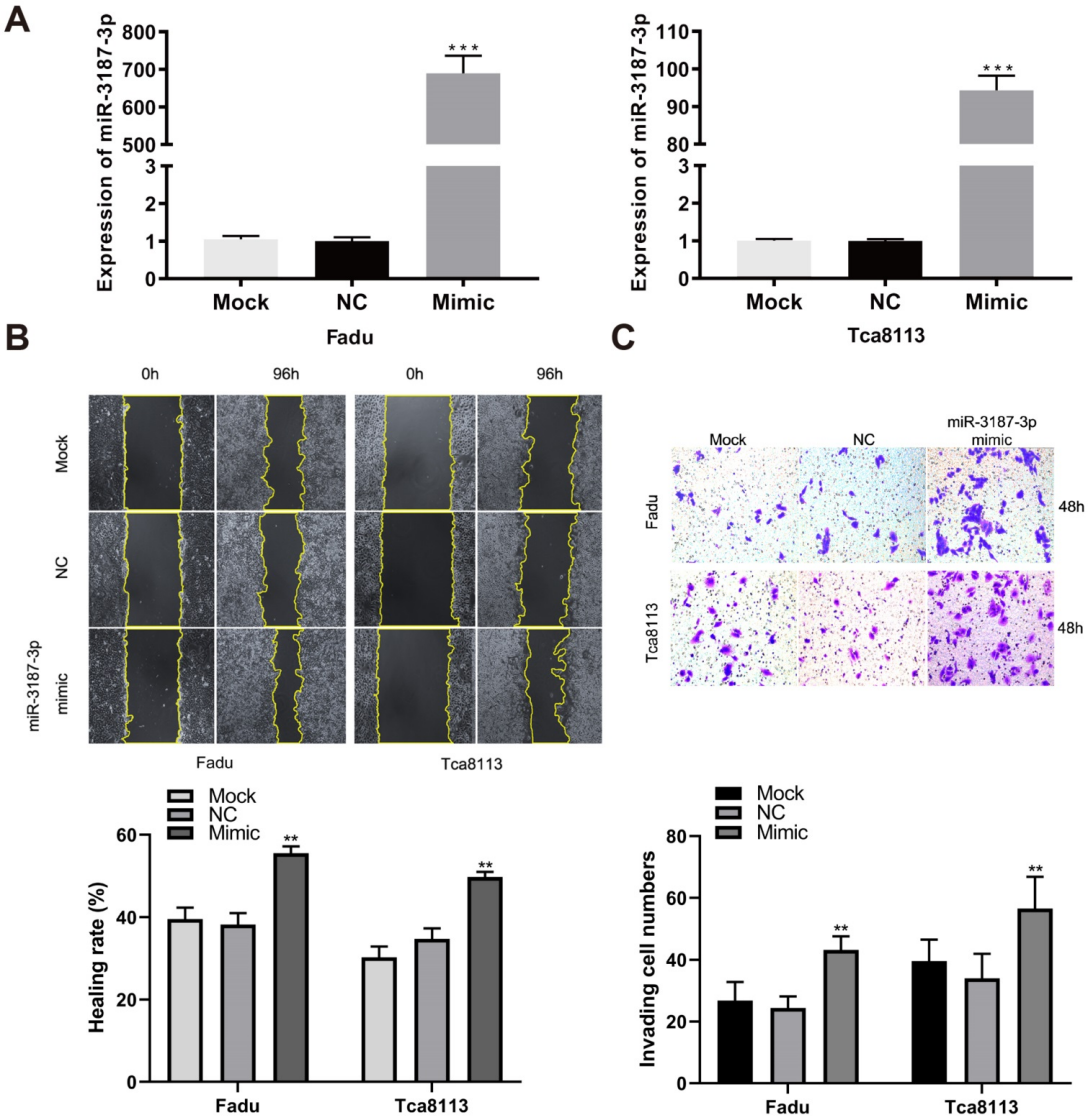
** Overexpression of miR-3187-3p promotes HNSCCs migration and invasion. A.** The expression of miR-3187-3p was measured using qRT-PCR after transfected with miR-3187-3p-mimic. **B.** The migration ability was assessed by the wound healing test, and the quantitative results of healing rate was determined. **C.** Representative images of transwell invasion assay for cells with different miR-3187-3p expression levels, and the invaded cells numbers were count. Data are presented as the mean ± SD. Student's unpaired t-test, **, P < 0.01; ***, P < 0.001.

To directly evaluate the tumor-promoting role of miR-3187-3p in HNSCCs, we quantified its expression in oral mucosa cell line DOK and HNSCCs cell lines, and found that the expression of miR-3187-3p was higher in HNSCCs cells than DOK cells (Supplementary Fig. S1). The relatively higher expression of miR-3187-3p cell lines (JHU011, Tu686) and relatively lower expression cell lines (Fadu, Tca8113) were selected for further loss and gain of function experiments, respectively. A significantly upregulated expression of miR-3187-3p was confirmed using qRT-PCR after transfection with miR-3187-3p mimics in Fadu and Tca8113 cell lines (Fig. 2A; P < 0.001). Wound-healing and transwell invasion assays were conducted to assess migration and invasion abilities of different HNSCCs cells. As shown in Fig. 2B, C, overexpression of miR-3187-3p significantly increased the migration and invasion capacities of cancer cells. Our results thus demonstrate that miR-3187-3p facilitates migration and invasion of HNSCCs cells.

### Downexpression of miR-3187-3p suppresses HNSCCs migration and invasion

**Figure 3:**
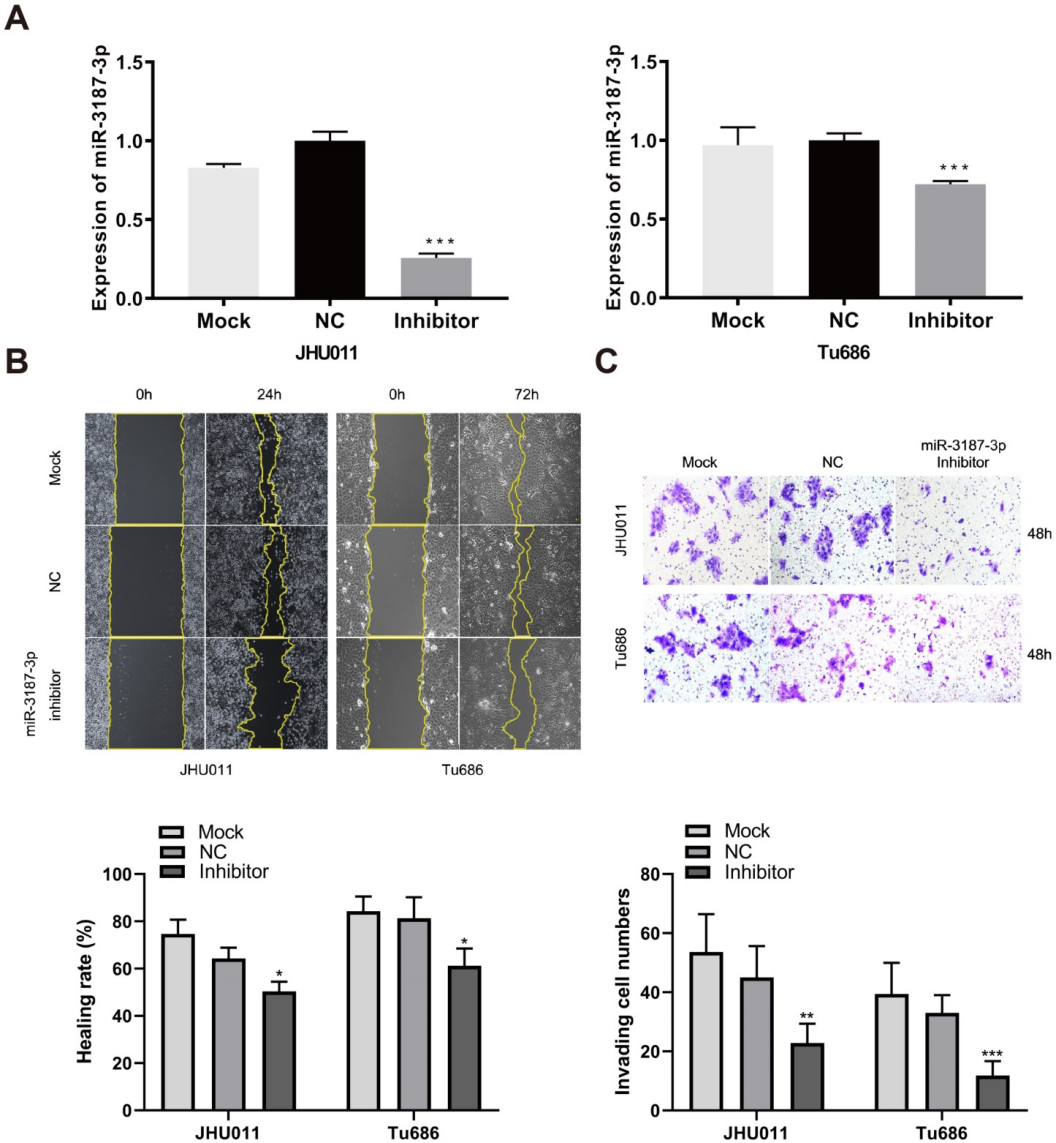
** Downexpression of miR-3187-3p suppresses HNSCCs migration and invasion. A.** The expression of miR-3187-3p in JHU011 and Tu686 cell lines was determined after transfection with miR-3187-3p inhibitor using qRT-PCR analyses. **B.** Wound healing tests in JHU011 and Tu686 cells indicated that cell migration ability was attenuated by the miR-3187-3p-inhibitor. **C.** Representative fields with invaded cells were obtained from transwell assays, and quantification of the invaded cells in different groups were compared. All data are represented as the mean ± SD. Student's unpaired t-test, *, P < 0.05; **, P < 0.01; ***, P < 0.001.

We conducted loss of function experiments to further confirm the role of miR-3187-3p in Tu686 and JHU011 HNSCCs cells. The expression of miR-3187-3p was successfully downregulated with inhibitors (Fig. 3A). As expected, the wound healing rates and successfully invaded cell numbers were reduced accordingly compared to the control (Fig. 3B, C), suggesting the negative regulation of tumor migratory and invasive ability by miR-3187-3p inhibition in HNSCCs.

### miR-3187-3p activates Wnt / β-catenin signaling pathway in HNSCCs

**Figure 4:**
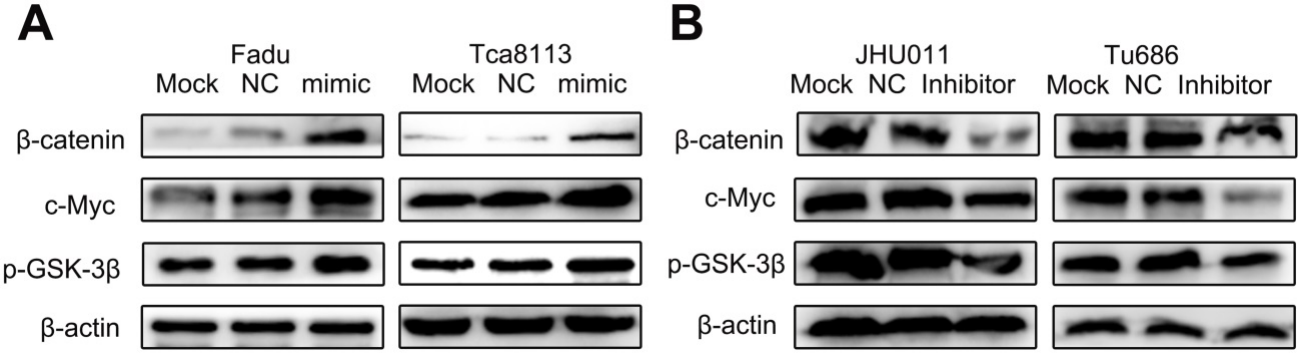
**miR-3187-3p activates Wnt / β-catenin signaling pathway in HNSCCs. A.** The protein levels of β-catenin、p-GSK-3β and c-Myc were analyzed by Western blot in Fadu and Tca8113 cells transfected with miR-3187-3p mimic. **B.** The protein levels of β-catenin、p-GSK-3β and c-Myc were analyzed by Western blotting in JHU011 and Tu686 cells transfected with miR-3187-3p inhibitor. β-actin was used as a loading control.

The Wnt / β-catenin pathway was a predominant oncogenic pathway, whose activation was the characteristic of multiple malignant biobehaviors, including tumor invasion and metastasis 19-23. In the study, we analyzed the relationship between levels of miR-3187-3p and biomarkers of Wnt / β-catenin pathway in HNSCCs cells by Western bolt. As shown in Fig. 4A, overexpressed miR-3187-3p significantly increased the protein expression of β-catenin, p-GSK-3β and c-Myc in Fadu and Tca8113 cell lines. Conversely, obvious downregulation of β-catenin, p-GSK-3β and c-Myc protein levels were observed by miR-3187-3p inhibition in JHU011 and Tu686 cell lines (Fig. 4B). These results indicated that miR-3187-3p activated the Wnt / β-catenin signaling pathway in HNSCCs.

### PER2 is a direct target of miR-3187-3p in HNSCCs

**Figure 5:**
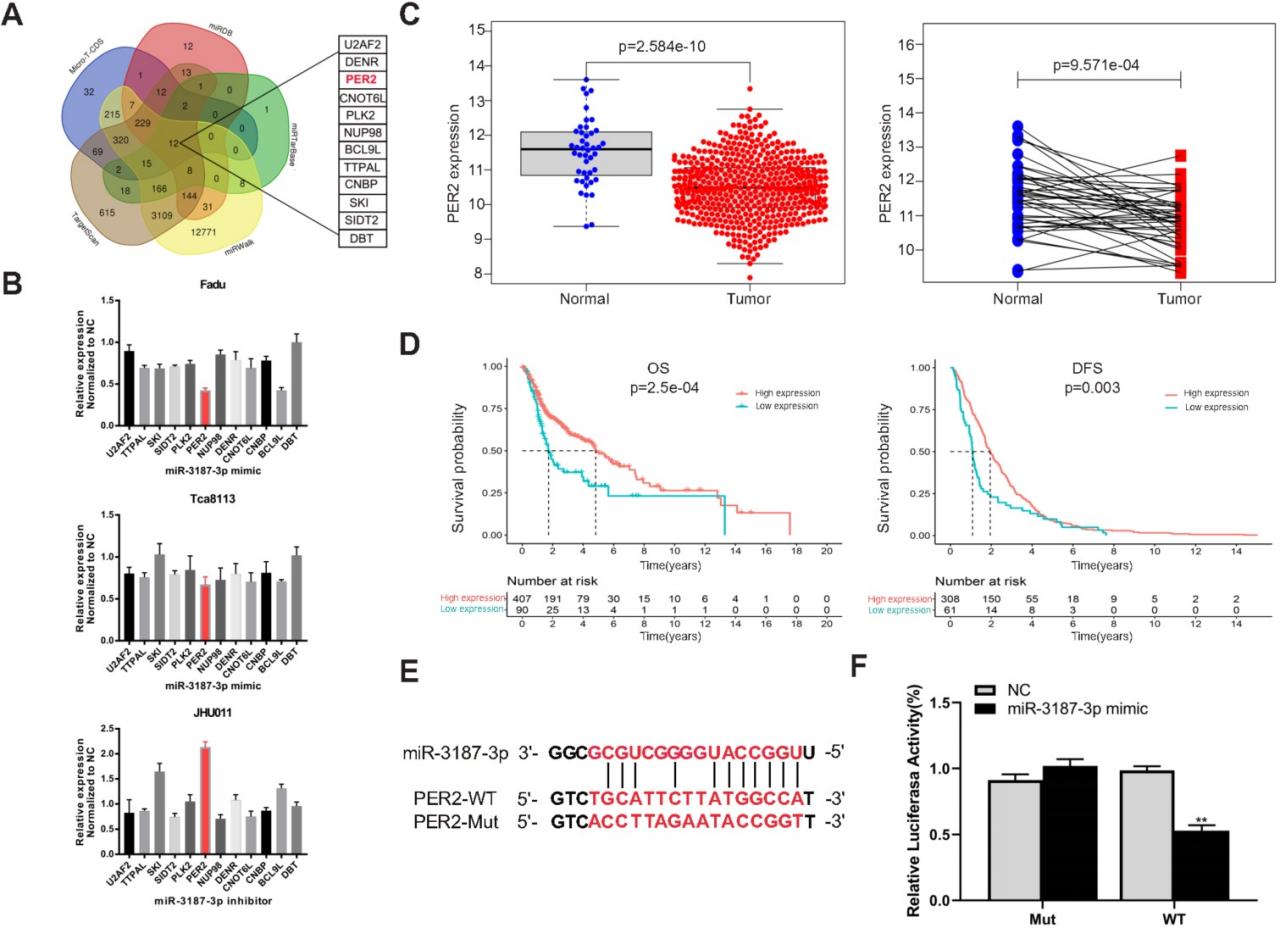
** PER2 is a direct target of miR-3187-3p in HNSCCs. A.** 12 candidate genes were identified through integrating the results of five algorithms (TargetScan, micro-T-CDS, miRDB, miRTarBase and miRWalk). **B.** Relative mRNA expression of candidate genes in Fadu, Tca8113 and JHU011 cell lines were detected using qRT-PCR after transfected with miR-3187-3p mimic or inhibitor. **C.** The expression of PER2 in 502 HNSCCs tissues and 44 adjacent normal tissues, and 43 paired HNSCCs and adjacent non-cancerous samples from the TCGA database were shown. **D.** Kaplan‐Meier curves of OS and DFS in patients with different expression levels of PER2 were shown (Best cutoff = 9.7). **E.** The binding sequence of miR-3187-3p and PER2 wild-type (WT), and the mutated (Mut) constructs of PER2 was shown. **F**. Luciferase reporter assays conducted in Fadu cell line demonstrated that PER2 was a direct binding target of miR-3187-3p. The luciferase activity was normalized to the corresponding NC. Data are presented as the mean ± SD. Student's unpaired t-test, **, P < 0.01.

In order to determine the potential target of miR-3187-3p, five online target gene prediction algorithms (TargetScan, micro-T-CDS, miRDB, miRTarBase and miRWalk) were used. As a result, 12 possible candidate target genes were gained, including PER2 (Fig. 5A). Then we upregulated and downregulated the expression of miR-3187-3p respectively in HNSCCs cell lines and identified PER2 as the most relevant gene (Fig. 5B). In addition, PER2 exhibited an opposite expression and prognosis style in contrast to miR-3187-3p according to the HNSCCs TCGA data analysis (Fig. 5C, D). Moreover, the luciferase reporter assay successfully validated that miR-3187-3p directly bound to the 3'UTR of PER2 as expected (Fig. 5E, F). These data demonstrated that PER2 is a direct target of miR-3187-3p in HNSCCs.

### PER2 mediates the effect of miR-3187-3p on HNSCCs migration and invasion

**Figure 6:**
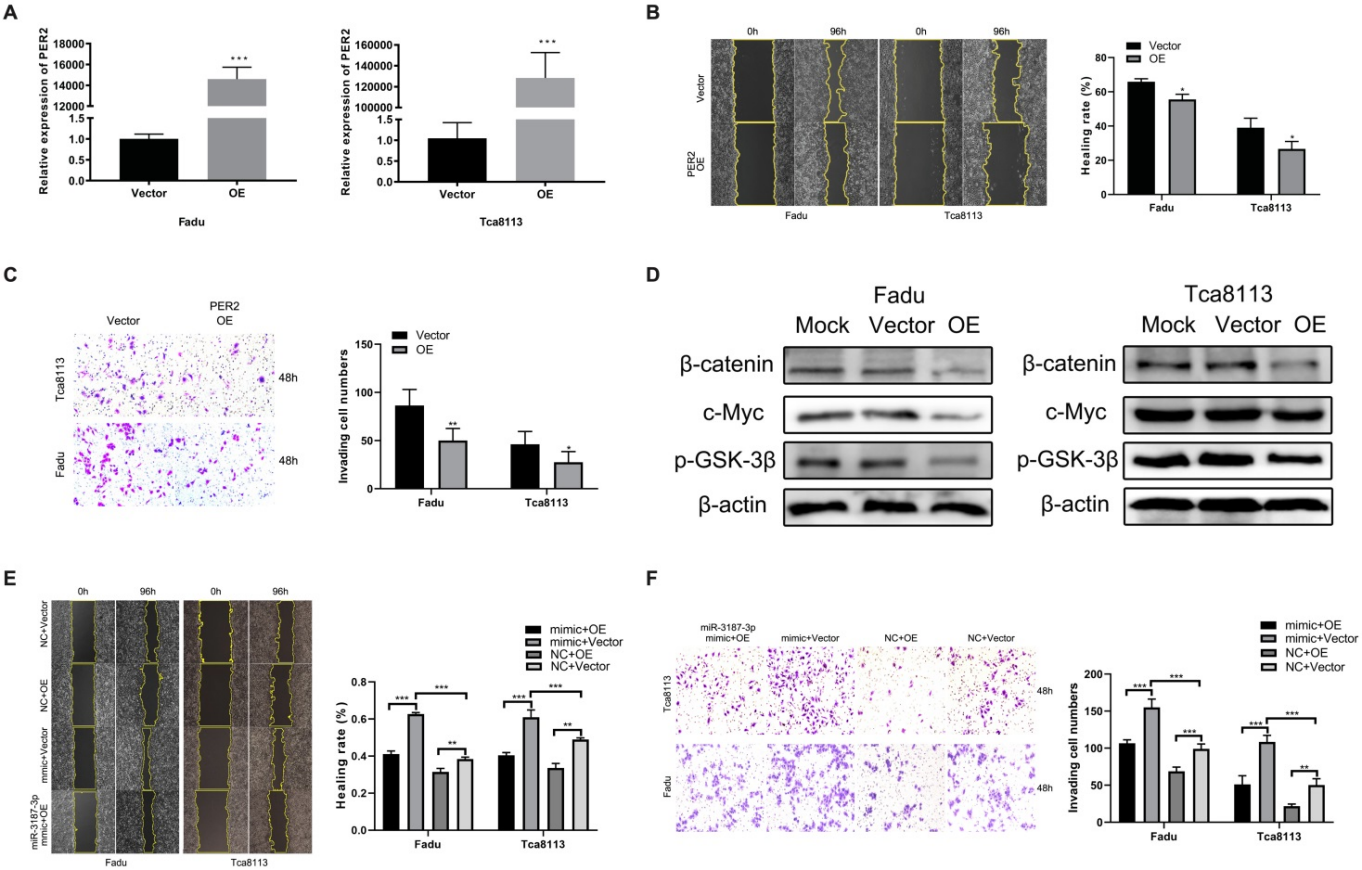
** PER2 mediates the effect of miR-3187-3p on HNSCCs migration and invasion. A.** PER2 mRNA expression significantly upregulated in Fadu and Tca8113 cell lines after transfection of PER2 plasmids. **B, C.** Following PER2 overexpression in Fadu and Tca8113 cells, wound-healing (B) and transwell (C) experiment were carried out. **D.** Western blotting showed that the expression of β-catenin, p-GSK-3β and c-Myc obviously downregulated in Fadu and Tca8113 cells transfected with PER2 plasmids. **E, F.** Fadu and Tca8113 Cells transfected with miR-3187-3p mimic or NC were subsequently disposed with PER2 or empty plasmid. Wound healing (E) and transwell staining (F) were evaluated in both cell lines. OE, PER2 overexpression. Data are presented as the mean ± SD. Student's unpaired t-test, *, P < 0.05; **, P < 0.01; ***, P < 0.001.

To ascertain if PER2 is functionally involved in HNSCCs migration and invasion mediated by miR-3187-3p, initially, the Fadu and Tca8113 cell lines were transfected with PER2 plasmids and an apparent PER2 overexpression was observed subsequently in both cell lines (Fig. 6A).Then, we performed wound healing and transwell assay to evaluate the changes in invasion and migration ability after upregulated PER2 expression in HNSCCs cell lines and the results indicated that upregulation of PER2 significantly inhibited migration and invasion in Fadu and Tca8113 cells (Fig. 6B, C). Apart from that, PER2 promotion also obviously decreased the protein levels of β-catenin, p-GSK-3β and c-Myc in cell lines described above (Fig. 6D). Finally, rescue experiments were conducted to confirm the role of PER2 on the miR-3187-3p-modulated migration and invasion in HNSCCs. PER2 plasmids were next transfected to Fadu and Tca8113 cells to antagonize the miR-3187-3p-mimic-induced hypoactive expression of PER2. As a result, the promotion effect of miR-3187-3p on HNSCCs migration and invasion were relieved by PER2 (Fig. 6E, F). Totally, our data clearly demonstrated that PER2 was involved in miR-3187-3p-mediated migration and invasion.

## Discussion

In the study, we identified that miR-3187-3p, overexpressed in tumor tissue, was related to the aggressive clinical features and poor prognosis of HNSCCs patients in data from TCGA, GEO and our tissue samples cohort. Further a series of *in vitro* experiments confirmed that miR-3187-3p promoted the migration and invasion of HNSCCs through targeting PER2, accompanied by the activation of Wnt / β-catenin signaling pathway, which deepened our understanding of the complicated mechanisms of migration and invasion in HNSCCs.

The latest data showed that HNSCCs was the seventh most common cancer in the world, causing approximately 450,000 deaths per year and tumor invasion and metastasis accounted for the primary causes of poor survival in HNSCCs patients 24. Besides, a pile of literatures had verified that miRNAs were concerned with the invasion and metastasis of diverse cancers 25-28, including HNSCCs 29-32.

Previously, we screened and identified an important N stage‐related miRNA, miR-3187-3p, in HNSCCs by bioinformatic analysis 16, and miR-3187-3p has been confirmed to be a cancer-promoting in several other cancers. For example, miR-3187-3p could promote colorectal cancer proliferation through targeting ASXL1 33, and upregulated miR-3187-3p expression was associated with enhanced tumor migration and invasion in non-small cell lung cancer 18, and salivary miR-3187-3p could facilitate tumor immune escape in melanoma 34. Our current study suggested that miR-3187-3p could enhance HNSCCs migration and invasion *in vitro* with the activation of Wnt / β-catenin pathway, which might be a novel potential therapeutic targets for improvement of HNSCCs patient outcomes.

Wnt / β-catenin signaling pathway was one of the key pathway in regulating cell proliferation and differentiation, playing a critical role in tumor invasion and metastasis 19, 21, 22, 35. β-Catenin was the core signal transduction in this signaling pathway, activation of which would give rise to high expression of downstream target genes 36. GSK-3β was a main inhibitory protein of the Wnt / β-catenin pathway, causing the degradation by ubiquitination of β-catenin. The phosphorylation of GSK-3β (p-GSK-3β) would lead to its inactivation, which contributed to the accumulation of β-Catenin and subsequently activation of downstream target genes, including c-Myc 23. C-Myc was a proto‐oncogene and had been reported to participating in the regulation of tumor progression and invasion in oral squamous cell carcinoma 37. Therefore, in our present study, miR-3187-3p may enhance the invasive potential of HNSCCs via the activation of Wnt / β-catenin signaling pathway with the upregulation of β-catenin, p-GSK-3β, and c-Myc.

MiRNAs exerted their functions by silencing the target genes, and it was well-established that a miRNA can bind and regulate multiple target genes, affecting various malignant behaviors of cancer cell 38. In the study, we identified PER2 as the direct target gene of miR-3187-3p and further analysis of TCGA data indicated that miR-3187-3p and PER2 exhibited a reversed expression state in HNSCCs samples, and high expression of PER2 correlated to a better prognosis in HNSCCs patients as well. PER2 was a well-known clock gene, regulating circadian rhythm in human beings, whose downregulation was related with tumor initiation and progression in various cancers, including HNSCCs 39-42. It has been reported that PER2 inhibited cell invasion and migration in human oral squamous cell carcinoma 43. In this study, we found that PER2 overexpression inhibited HNSCCs cells migration and invasion *in vitro*, showing that PER2 served as a negative regulator in HNSCCs metastasis, consistent with the previous reports. We also identified that upregulation of PER2 attenuated migration and invasion ability of HNSCCs induced by miR-3187-3p, implying that PER2 may be a main target of miR-3187-3p. Additionally, PER2 overexpression also proved to be positive correlated to the expression of Wnt / β-catenin pathway, which was compatible with previous report in glioblastoma 44.

In conclusion, our study support that high expression of miR-3187-3p is strongly linked to the malignant traits of HNSCCs. High expression of miR-3187-3p predicts a poor outcome in patients with HNSCCs. Moreover, a series of *in vitro* evidence suggest that miR-3187-3p can directly target tumor suppressor gene PER2, promoting migration and invasion of HNSCCs by activating the Wnt / β-catenin pathway. Overall, this study illustrates that the miR-3187-3p / PER2 axis may provide a novel promising molecular treatment strategy to subdue the progression of HNSCCs. In future studies, we will investigate how miR-3187-3p / PER2 axis is involved in the HNSCC metastasis in animal models and explore the potential value of this axis as a therapeutic window.

## Supplementary Material

Supplementary figure and table.

## Figures and Tables

**Table 1:** Correlations between miR-3187-3p expression and clinicopathological features in TCGA HNSCCs patients.

Clinical characteristics	Total (N)	ANOVA P-value	ANOVA FDR
Clinical M Status	497	6.21e-01	9.80e-01
Clinical N Status	500	***6.17e-05***	***4.73e-03***
Clinical Stage	508	9.93e-01	9.96e-01
Clinical T Status	506	9.79e-01	9.91e-01
Histologic Grade	499	3.85e-01	6.04e-01
Pathologic M Status	189	8.70e-01	9.58e-01
Pathologic N Status	422	***6.80e-07***	***5.71e-05***
Pathologic Stage	450	5.54e-01	9.48e-01
Pathologic T Status	460	3.05e-01	6.41e-01
Sex	522	5.92e-01	8.48e-01

Note: This table was obtained from the OncomiR (http://www.oncomir.org/). *P < 0.05 was considered to be statistically significant (in bold and italics).

**Table 2:** Correlations between miR-3187-3p expression and clinicopathological parameters in HNSCCs clinical samples.

Parameters	No. of patients	miR-3187-3p expression	*t* value	*P* value*
Age				
<59	41	0.79±0.60	0.522	0.603
≥59	42	0.73±0.34		
Gender				
Female	4	0.62±0.28	0.580	0.564
Male	79	0.77±0.51		
Smoking				
Yes	47	0.76±0.39	-0.033	0.974
No	36	0.76±0.62		
Histological grade				
G1 + G2	29	0.78±0.63	0.305	0.761
G3	54	0.75±0.41		
T classification				
T1	16	0.52±0.20	-2.179	***0.032***
T2+ T3 + T4	67	0.82±0.53		
Clinical stage				
I + II	36	0.54±0.21	-3.869	***<0.001***
III + IV	47	0.93±0.58		
Lymph node metastasis				
N0	54	0.60±0.30	-4.394	***<0.001***
N+	29	1.06±0.64		

*P < 0.05 was considered to be statistically significant (in bold and italics).
