# Supplementary material for: miR-3187-3p enhances migration and invasion by targeting PER2 in head and neck squamous cell carcinomas
Source: J Cancer. 2021 Jun 26;12(17):5231–40. doi: 10.7150/jca.58593 (PMC8317515; doi:10.7150/jca.58593)
Supplement: Supplementary file 1 — Supplementary figure and table. [file jcav12p5231s1.pdf]

Supplementary Figure S1. The basic expression of miR-3187-3p in DOK and HNSCCs cell lines.

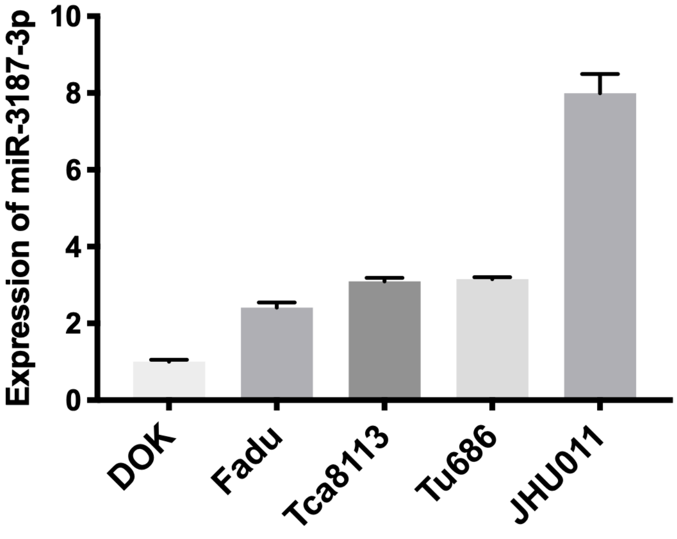

Supplementary Table S1. The sequences of primers

U2AF2	FORWARD	CGGCTGGAGAGGTGGAAGGAG
	REVERSE	GGAGGGCAGTGGGCAGGAG
DENR	FORWARD	ACCGCCTGGGTTTGTGAAATGG
	REVERSE	TGGCACTGTTCCTTGGGTCTCC
PER2	FORWARD	CTTATTCACTGCCCGTGTTTC
	REVERSE	GGAAGGAATAACTGGGTAGCAT
CNOT6L	FORWARD	CCCTCCACTCCTGCCTCTTGTC
	REVERSE	GCAACAGATCCCCGTCTTGGC
PLK2	FORWARD	GAGATCTCGCGGATTATCGTC
	REVERSE	CGGCGTAGACTTTGTTATTTGT
NUP98	FORWARD	GGGCTTGTTTACCTGGACTATA
	REVERSE	CACTTTGATGTGTAACTGCTCC
BCL9L	FORWARD	GAAGGAGGAGCAGGAGCAGGAG
	REVERSE	CGGGCAGTGGCGGGAGAG
TTPAL	FORWARD	GAAAGTGACTCTCTGAGAACCA
	REVERSE	CACATCTCGAAGTCTCCATTCC
CNBP	FORWARD	GAGGTTTCCAGTTTGTTTCCTC
	REVERSE	CCTCCTGAAGATCACAATCCTT
SIDT2	FORWARD	TCGTCTTCTCCATCATTCACAT
	REVERSE	AAATCATTGGGGCGCATGATAA
SKI	FORWARD	CTCATCACCAAGACGGACG
	REVERSE	AGCAGCCCCTTACACTTG
DBT	FORWARD	AATGGTCAAGACTATGTCTGCA
	REVERSE	AATTCTTCTCGGAGCTTAACCA
GAPHD	FORWARD	TCCAAAATCAAGTGGGGCGA
	REVERSE	AGTAGAGGCAGGGATGATGT
